# Drug Transport System Based on Phospholipid Nanoparticles: Production Technology and Characteristics

**DOI:** 10.3390/pharmaceutics14112522

**Published:** 2022-11-19

**Authors:** Elena G. Tikhonova, Maxim A. Sanzhakov, Yulia A. Tereshkina, Lyubov V. Kostryukova, Yulia Yu. Khudoklinova, Nadezhda A. Orlova, Daria V. Bobrova, Olga M. Ipatova

**Affiliations:** Institute of Biomedical Chemistry, 10 Pogodinskaya St., 119121 Moscow, Russia

**Keywords:** soy phosphatidylcholine, phospholipid nanoparticles, dosage form, dispersion, technological factors of lyophilization

## Abstract

One of the current trends in modern pharmaceuticals is the supply of drugs by transport systems. The use of delivery systems allows to increase the therapeutic efficacy, tolerability, and safety of drug therapy. Liposomes, polymer nanoparticles, carbon nanoparticles, blood cells, metal nanoparticles, oxides, etc., are used as transport systems. This work is aimed at obtaining a finished technological product based on soy phospholipids with particle size in the nanometer range and reproducible characteristics (size, charge). For this purpose, we carried out investigations to select the optimal conditions of technological process. The developed technology makes it possible to obtain phospholipid nanoparticles without the use of any solubilizers and/or surfactants, which increases its practical relevance for further work. The versatility of the technology is demonstrated by the example of incorporation of drugs of various chemical nature and pharmacotherapeutic groups.

## 1. Introduction

Much attention is now being paid to technologies for the development of drug transport systems [1,2]. This approach is beneficial both medically and economically. Transport systems increase the solubility of hydrophobic drugs, improve pharmacokinetics and their ability to penetrate cells, while many medications gain the ability to penetrate physiological barriers [3,4,5,6]. Drug transport/delivery systems contribute to an increase in therapeutic effectiveness, which allows to reduce the dose or frequency of medication and shortens the duration of treatment.

Recently, nanoparticles of various nature have been used as transport nanosystems, for example, systems based on synthetic polymers, dendrimers, and natural materials (alginate, chitosan, silicon, silver, etc.) [7]. However, plant phospholipid-based transport/delivery systems are of particular interest, since they are biodegradable, do not cause allergic reactions, and the surface of such nanoparticles can be easily modified to impart certain properties, including an increase in drug bioavailability. Incorporation of drugs in the composition of nanoparticles (in particular, liposomes) protects the drug from premature degradation in the bloodstream, reduces its clearance, and makes it possible to use lower doses [8].

The particle diameter of existing liposomal preparations is usually 100–400 nm. The possibility of obtaining extremely small particles (less than 100 nm) allows (1) to protect them from “capturing” by the reticuloendothelial system (RES), (2) increase their stability and circulation time in the blood, and (3) ensure the delivery of the drug into the cell. Indeed, the smaller the particle size, the more pronounced their optimizing effect on drug pharmacokinetics and the effectiveness of its penetration in organs and tissues [9]. Many well-known liposomal preparations are nanoemulsions, less often lyophilized powder. However, the disadvantage of liquid drug form is a short shelf life, which incurs inconveniences for its practical use [10]. 

The development of efficient transport systems, including those based on plant phospholipids, is primarily associated with overcoming various technological problems. According to its physicochemical characteristics, a phospholipid raw material is a solid, fat-like, water-insoluble substance. Thus, special attention is paid to the development of methods for preparation of homogeneous ultrafine emulsion, followed by its lyophilic drying and obtaining a dry lyophilized powder stable during storage and transportation. In this regard, the main technological tasks that need to be solved are as follows: selection of optimal method for obtaining phospholipid nanoparticles for subsequent incorporation of drugs; elaboration of the conditions for lyophilic drying of homogeneous ultrafine nanoemulsion, which, during subsequent rehydration of the resulting powder, ensure the formation of a nanoemulsion consisting of standard-sized particles. 

To date, there are three main methods for obtaining phospholipid nanoparticles (micelles/liposomes): (1) Treatment of a coarse phospholipid emulsion in water using ultrasound (sound disintegration); (2) extrusion of coarse phospholipid emulsion in water; and (3) homogenization of coarse phospholipid emulsion in water under high pressure (1000–1500 bar).

The method of sound disintegration has not been widely used, since it has a number of disadvantages. For example, the emulsions thus obtained are often contaminated with titanium particles (the material of the voicing tip). Moreover, during the treatment with ultrasound, the solution is subjected to strong heating and unlimited contact with the environment, which leads to the oxidation of phospholipids. A major factor limiting the application of ultrasound is the need to use energy-intensive high-power installations to obtain small phospholipid particles. Thus, this method is usually considered as rough, and is mainly used in scientific research [11]. 

To obtain fine phospholipid emulsions, the method of multiple pressing (extrusion method) of a dispersed phase substance through thin holes (pores) of the membrane into a dispersion medium under high pressure is used. However, this method has not found distribution on an industrial scale.

High-pressure homogenization is a widely used method to obtain phospholipid nanoparticles, including liposomes. It can be divided into slit homogenization and microfluidization. Homogenization is carried out either using a piston system in which a coarse emulsion is forced at a pressure of 1000–1500 atm through a valve with an adjustable gap (slit homogenization), or through ceramic chambers of a certain geometry, with a diameter of 75 and 200 microns (microfluidization) [12]. 

Another way of dispersing lipids into an aqueous medium is the use of detergents, in the presence of which spontaneous formation of micelles/liposomes occurs. At the end of this process, the detergent is gradually removed from the solution, for example, by dialysis. However, the list of detergents approved for use in pharmacology is very limited, and the method itself is not sufficiently technological to be used on an industrial scale [13]. 

The production of fine phospholipid particles is also possible using the method of “soft” homogenization (multiple repetition of freeze–thaw cycles). This technique is usually used in combination with other homogenization methods, but it is also unsuitable for industrial production [14]. 

This article presents the results of research on the development of phospholipid transport nanosystem (PhTNS) and PhTNS-based drug compositions using high pressure homogenization.

## 2. Materials and Methods

### 2.1. Materials

The following materials were used: soy phospholipid Lipoid S100 (Lipoid, Ludwigshafen, Germany) with a phosphatidylcholine content of ≥94%, maltose monohydrate (Merck, Darmstadt, Germany), drugs: doxorubicin (OAO Omutninsk Scientific Experimental-Industrial Base, Vostochnyi, Russia), indomethacin (Huzhou Synthetic Pharmaceutical Factory, Huzhou, China), umifenovir (ABC Farmaceutici, Canton Moretti, Italy), diclofenac sodium (Henan Dongtai Pharm Co., Ltd., Henan, China), budesonide (Long-Sheng Ltd., Beijing, China), prednisolone synthesized at the Center for Bioengineering of the Russian Academy of Sciences (Moscow, Russia), di-N-methyl-D-glucamine salt of chlorine e6, with 50% content of chlorine e6 (Ivanovo State University of Chemistry and Technology, Ivanovo, Russia).

Rectified ethyl alcohol 96% (Konstanta-Farm M, Moscow, Russia), methanol for HPLC, acetonitrile for HPLC gradient analysis (Scientific UK Ltd., Stoke-on-Trent, UK), trifluoroacetic acid (TFA) ≥ 99% (Acros organics, Fair Lawn, NJ, USA), distilled or purified water (Milli-Q, Darmstadt, Germany), a kit for enzymatic colorimetric determination of phospholipids (Sentinel Ch. SpA, Milano, Italy), water for injection (LLC East Farm, Ussuriysk, Russia), phosphate buffered saline (PanEco, Moscow, Russia).

### 2.2. Methods

#### 2.2.1. Preparation of Phospholipid Nanoparticles

Lipoid S100 (25.0 g) was suspended in an aqueous solution of one of the sugars (100 g/500 mL) to obtain a primary (coarse) emulsion in the ratio of 1:4 phospholipid:sugar. The coarse emulsion was poured into the receiving tank of the homogenizer APV 2000 (APV manufacturing Poland Sp. z.o.o, Bydgoszcz, Poland) (method A) or into the receiving tank of the microfluidizer M110EH30K, Microfluidics, Westwood, MA, USA (method B) and homogenized in cyclic mode, varying temperature, pressure, and number of cycles. After each cycle, sampling was carried out to control the particle size. The resulting ultrathin emulsion was filtered using the YY30 090 00 filtration unit (Millipore Corporation, Burlington, MA, USA), passing through a fiberglass prefilter with a pore size of 1 micron and a membrane filter with a pore size of 0.22 nm. The filtrate (10 mL of each sample) was poured into 20 mL vials and dried using the freeze drier Virtis AdVantage XL (SP Scientific, Warminster, PA, USA).

#### 2.2.2. Determination of Particle Size

Particle size was determined using the Zetasizer Nano ZS laser correlation spectrometer (Malvern Instruments Ltd., Malvern, UK) using dynamic light scattering. Three measurements were carried out, the result in the form of polydisperse distribution of particles by volume was averaged.

Micrographs of phospholipid nanoparticles were prepared using a Jeol 100 CX electron microscope (Jeol, Tokyo, Japan) at a magnification of 20,000 using negative contrast with a 1% aqueous solution of uranyl acetate.

#### 2.2.3. Determination of the ζ-Potential Value

ζ-potential was measured using electrophoretic light scattering on the Zetasizer Nano ZS analyzer (Malvern Instruments Ltd., Malvern, UK). 

#### 2.2.4. Incorporation of Drug into Phospholipid Nanoparticles

Lipoid S 100 (5 g) was dispersed in an aqueous maltose solution (20 g/200 mL). When stirring, one of the following drugs was added as a dry powder: doxorubicin hydrochloride, indomethacin, umifenovir, diclofenac sodium, budesonide, prednisolone, chlorine e6 N-methyl-D-glucamine salt in the ratio of 10:1 phospholipid:drug.

The resulting coarse emulsion was diluted with water to a volume of 500 mL and subjected to several (5–7) homogenization cycles on the microfluidizer at a pressure of 1000 bar and a temperature of 42–45 °C. The resulting ultrathin emulsion was then successively filtered through a fiberglass prefilter with a pore size of 1 micron and a membrane filter with a pore size of 0.22 nm. Each sample of filtrate (10 mL) was poured into 20 mL vials and dried using the freeze drier. 

#### 2.2.5. Measurement of the Percentage of a Drug in Nanoparticles 

The percentage of a drug in nanoparticles was determined using the HPLC method. For all drugs, a device was used with a G1315B spectrophotometric detector (Agilent Technologies, Santa Clara, CA, USA), Eclipse XDB-C18 column (4.6 mm × 150 mm, 5 μm) (Agilent Technologies). Mobile phase: 0.1% aqueous solution of trifluoroacetic acid (A) and 0.1% solution of trifluoroacetic acid in acetonitrile (B). The flow rate of the mobile phase was 0.5 mL/min and the injection volume was 10 µL. The chromatography conditions for particular drugs are as follows (Table 1):

The percentage of a drug in nanoparticles was calculated by the formula:

(C − C_f_) × 100/C,

where C is the concentration of drug in the test solution; C_f_ is the concentration of drug in the filtrate.

#### 2.2.6. Drug Release In Vitro

The release of drugs embedded in PhTNS (doxorubicin, indometacin, umifenovir, diclofenac, budesonid, prednisolon, chlorin e6) was evaluated using a dialysis membrane method (3.5 kDa). Briefly, 1 mL of test sample (the drug concentration was 1 mg/mL) in the dialysis bags was placed in 25 mL of phosphate buffered saline (PBS) (pH 7.4) and incubated with stirring at 37 °C and 100 rpm on a Grant OLS 200 shaker water bath (Grant Instruments (Cambridge) Ltd., Shepreth, UK). The aliquots of the supernatant (1 mL) were sampled at certain intervals for each variant (0.25; 0.5; 1; 2; 5; 8; 24; 48, and 72 h). After each sampling, an equal amount of PBS was added. The concentration was determined using a spectrophotometer Agilent 8453 spectrophotometer (Agilent Technologies, Waldbronn, Germany) at the appropriate wavelength (254 nm (Doxorubicin), 268 nm (Indomethacin), 486 nm (Umifenovir), 276 nm (Diclofenac), 248 nm (Prednisolon), 405 nm (Chlorin e6)). The rate of drug release was calculated by dividing the concentration of each drug (released from drug in phospholipid nanoparticle) at a certain time by the initial concentration of corresponding drug in phospholipid nanoparticle [15].

#### 2.2.7. Determination of Moisture Content

Residual moisture was determined on the automatic titrator Mettler toledo DL31 (Mettler-Toledo International Inc., Greifensee, Switzerland) using Hydranal Solvent, two-component titrant Hydranal Titrant 5 and Hydranal Eichstandard 5.00 water standard (Fluka, Seelze, Germany).

#### 2.2.8. Statistics

We used the Student’s criterion (*p* ≤ 0.05) to evaluate the reliability of differences in the average particle size for two samples. The figures and the tables show the data as mean ± standard error of the mean. 

## 3. Results and Discussion

Analysis of literature data and our own experience with phospholipids of plant origin suggest that homogenization under high pressure (1000–1200 atm.), including slit homogenization and/or microfluidization, are the most technologically advanced methods of obtaining phospholipid particles for their use as a drug transport system [16,17,18,19]. Among the advantages are high productivity and minimal oxidation of phospholipids during processing. These technologies ensure reproducibility and standardization of the resulting particles by size. Application of high-pressure homogenizer and/or microfluidizer allows performing experiments under aseptic conditions, with constant temperature and pressure control.

The selection of conditions for obtaining phospholipid nanoemulsion using high-pressure homogenization was carried out using the homogenizer and the microfluidizer.

### 3.1. Optimization of High-Pressure Homogenization

**Figure 1:**
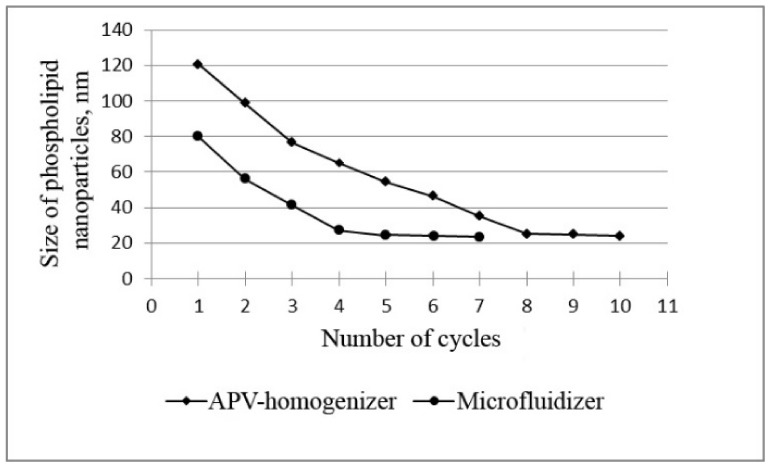
Dependence of the size of phospholipid nanoparticles on the number of high-pressure homogenization cycles using the APV homogenizer and microfluidizer at T = 45 °C and P = 1000 bar.

At the initial stage, a series of experiments was carried out to determine the effect of the number of homogenization cycles on the nanoparticle size (Figure 1), with a pressure of 1000 bar and a temperature of 45 °C. 

The data in Figure 1 prove that, when phospholipids are homogenized using the microfluidizer, a nanoemulsion with the smallest particle size of phospholipids (less than 30 nm) is formed after five cycles. Similar results were obtained using a slit APV homogenizer, but this required a longer processing time—8 homogenization cycles. It is also important to note that the nanoemulsion obtained with the microfluidizer (5 cycles) was more homogeneous: the polydispersity index (PI) was slightly lower (0.554) compared to the PI of samples obtained with the APV homogenizer (0.646). The polydisperse analysis confirms this conclusion: 94% of particles with a diameter of (24 ± 4) nm was found in the nanoemulsion obtained using the microfluidizer, whereas in the nanoemulsion obtained using the APV homogenizer, only 88% of the particles had a diameter of (25 ± 6) nm.

Thus, these data show that five cycles of microfluidizer and eight cycles of slit homogenizer are sufficient to obtain very fine phospholipid nanoparticles. 

### 3.2. Influence of Other Parameters on the Size of Phospholipid Nanoparticles

**Figure 2:**
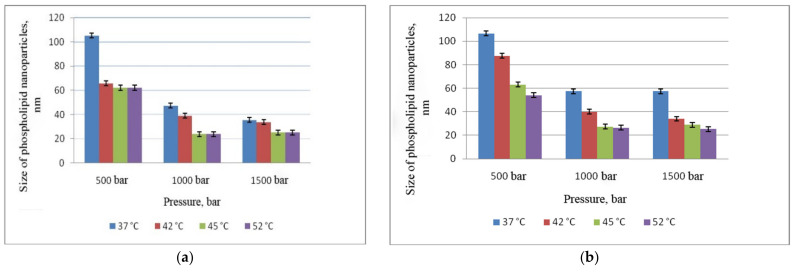
Dependence of phospholipid nanoparticle size on pressure and temperature: (**a**) microfluidizer; (**b**) APV-homogenizer.

At the next stage, we evaluated the influence of temperature and pressure on the size of phospholipid nanoparticles (Figure 2). At the same time, the optimal number of cycles was used to obtain nanoemulsions (five cycles of microfluidization and eight cycles of slit homogenization, Section 3.1).

Figure 2 shows that at a pressure of 500 bar and T = 37 °C, the particle size obtained both using the microfluidizer and APV homogenizer exceeded 100 nm. An increase in the temperature led to a decrease in the size of phospholipid particles to 60 nm. The smallest size (less than 30 nm) was obtained at a temperature of 45 °C and a pressure of 1000 bar by both methods (A and B). A further increase in pressure up to 1500 bar did not lead to a significant reduction in size and was accompanied by oxidation of phospholipids both in the case of APV homogenizer and when using the microfluidizer.

Thus, the optimal parameters for obtaining a phospholipid emulsion with an extremely small size of nanoparticles are as follows: the number of cycles 8 when using a slit homogenizer and 5 when using a microfluidizer, T = 45 °C, P = 1000 bar. It should be noted that application of microfluidizer involves less energy consumption, with more accurate and convenient control of technological parameters.

### 3.3. Standardization of Particle Size and Sterilizing Filtration

Standardization of particle size is a crucial problem in the production of phospholipid nanoparticles on an industrial scale, in particular, liposomes. It is known that large lipid agglomerates in preparations for intravenous administration are unacceptable. In addition, phospholipids are thermolabile and easily oxidizing compounds, therefore, thermal sterilization cannot be used for medicines obtained on their basis. 

To solve the above problems, we applied the method of standardizing filtration through glass fiber prefilters with a pore size of 1.0 micron, followed by sterilizing filtration through membranes with a pore size of 0.22 micron. To do this, the secondary emulsion was fed to filters fixed in the filter holder using a peristaltic pump with an adjustable head rotation speed (XX80EL004, Millipore, Molsheim, France). The maximum rotation speed provided a filtration rate of up to 1000 mL/min. At the same time, at a high filtration rate, the filter pores clogged faster, and the process had to be stopped to replace the membrane filters. The experience showed that a good filtration rate could also be achieved with a filtration rate of 30% of the maximum speed. Filtration of the secondary emulsion at a rate of 300 mL/min gave satisfactory results. Therefore, 300 mL/min was chosen as an optimal filtration rate.

### 3.4. Selection of a Cryoprotectant and Optimization of Freeze Drying Process

The drying process is of key importance in obtaining PhTNS and PhTNS-based medicinal compositions in the form of a dry powder. This is due to the instability of phospholipid emulsions. Phospholipids themselves can be easily oxidized and hydrolyzed, and the resulting nanoparticles are thermolabile and prone to aggregation. 

Among various drying methods widely used in the pharmaceutical industry (vacuum, spray, fluidized or gushing layer and other hydrodynamic modes), vacuum freeze drying has become the most common.

Freeze-drying makes it possible to preserve the original structure and properties of the preparations and avoid the disadvantages of thermal processing methods. Due to low temperatures, the original properties of the product are completely preserved [20]. 

Therefore, we have developed and optimized the technological conditions of lyophilization of phospholipid nanoemulsions to obtain a dry powder of phospholipid nanoparticles that are stable during long-term storage and retain their physical and chemical properties during rehydration. 

It should also be noted that during lyophilization, phospholipid structures are exposed to many destabilizing factors. Thus, freezing can lead to physical damage of phospholipid aggregates by ice crystals, removal of surrounding water can initiate the processes of destruction and fusion of phospholipid particles. In addition, rehydration can cause undesirable phase transitions in phospholipid structures. 

Cryoprotectants are known to be used during freeze-drying to stabilize the supramolecular structures obtained. In addition, a cryoprotectant also determines the speed and duration of the drying process [21]. Sugars are mainly used as cryoprotectants when drying phospholipid particles.

**Figure 3:**
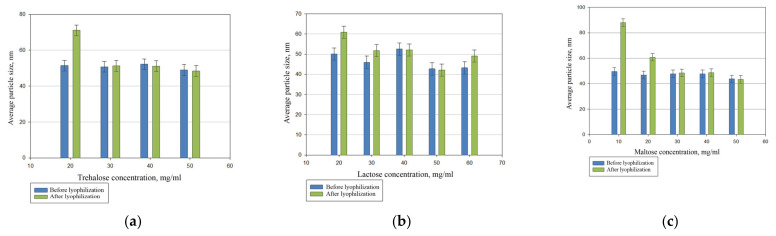
The effect of trehalose (**a**), lactose (**b**), and maltose (**c**) content on the reproduction of the particle size of PhTNS after rehydration.

We compared the cryoprotective properties of the most commonly used sugars: maltose, lactose, and trehalose (Figure 3).

The comparative studies showed that maltose, lactose, and trehalose have comparable properties. However, we applied maltose as a cryoprotectant since it is often used in the pharmaceutical industry. 

In addition, lyophilization temperature regime may change the properties of the drying emulsion. Thus, its optimization is necessary. A freeze-drying process comprises three stages: (1) freezing stage; (2) removal of frozen water from the product (sublimation stage); (3) removal of unfrozen (bound) water by heating to an acceptable level (secondary drying step). 

The amount of residual moisture significantly affects the stability of the dry preparation during storage. At the end of the sublimation stage, the moisture content in the product did not exceed 6%. During the secondary drying process, the moisture content decreased to a level of ≤3%. 

It is believed that during freezing and lyophilization, the temperature of the drying material should be at least 10 °C below the glass transition temperature (Tg). In this case, the phospholipid nanoparticles retain their stability. Experimentally, we have found that Tg is about −40 °C for medicinal compositions containing about 87% water, 10% maltose, 2.5% phospholipids (Lipoid S100), and less than 1% of the drug and other components. Therefore, when freezing, the temperature of the product had to be brought at least 50 °C below zero, keeping it frozen for several hours before lyophilization. 

**Figure 4:**
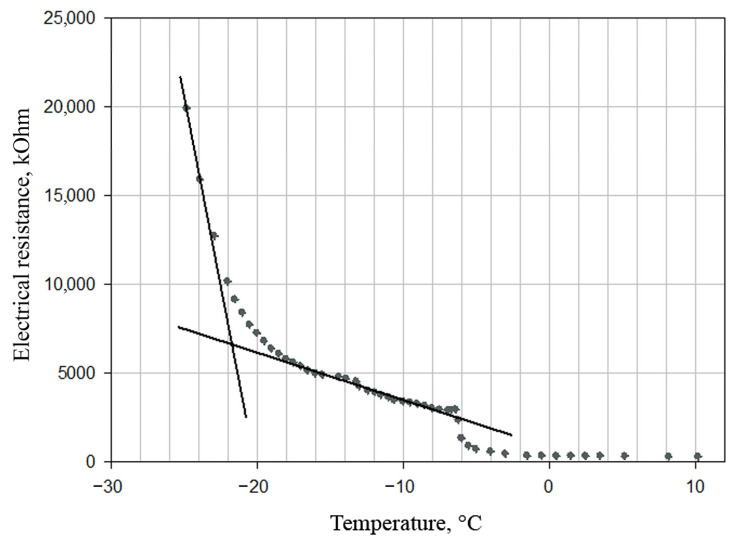
Dependence of the electrical resistance of the emulsion on temperature during its defrosting. The temperature at the intersection of the tangents corresponds to that at the eutectic point −21.5 °C.

However, lyophilization at such temperature (−50 °C) increases the total drying time and, accordingly, energy consumption. Experimental studies have shown that during lyophilization of emulsion based on phospholipid nanoparticles, it is sufficient to maintain a temperature below the eutectic point, i.e., the highest temperature at which complete crystallization of the drying material occurs. To determine the eutectic point, we analyzed the dependence of nanoemulsion electrical resistance on temperature during the defrosting process (Figure 4).

Figure 4 shows that the eutectic temperature of the sample—the intersection of the tangents—is −21.5 °C. Therefore, the optimal condition for lyophilization is a temperature below −21.5 °C. In order to not exceed the temperature, the process is carried out at a constant speed, i.e., with a constant power supply. To do this, the temperature of a heat transfer agent (i.e., freeze-drying shelves) is maintained constant. 

The freeze-drying process was optimized based on the analysis of thermograms. The main adjustable parameter is the freeze-drying shelf temperature. It was found that a gradual increase in its temperature led to a significant increase in the eutectic point before the end of the sublimation stage. This was the reason for partial melting of the vial’s contents and the evidence of boiling in the drying material. To obtain a high-quality product, it was proposed to maintain the shelf temperature at a constant level. At the same time, it was found that the maximum shelf temperature at the sublimation stage, at which the quality of the drying material is preserved, equals 20 °C. Thus, the optimal shelf temperature at the secondary drying stage will be 40 °C.

Thus, we determined the following optimal technological conditions of the drying stage at the pilot plant:

- Freezing of the product to a temperature of −40 °C;
- Reduction of the temperature to −50 °C by additional two-hours of freezing;
- Lyophilization at a product temperature −21.5 °C and a shelf temperature of 20 °C for 500–1000 min (depending on the amount of drying material);
- Secondary drying step at a shelf temperature of 40 °C for 500–1000 min (depending on the amount of drying material).

### 3.5. Physical Properties of Phospholipid Nanoparticles

The particle size and zeta potential were measured after freeze drying, i.e., in the dehydrated samples.

#### 3.5.1. Laser Correlation Spectroscopy (Dynamic Light Scattering)

Dynamic light scattering (DLS) furnishes important information on the structure and dynamic properties of nanoparticles in solution [22].

**Figure 5:**
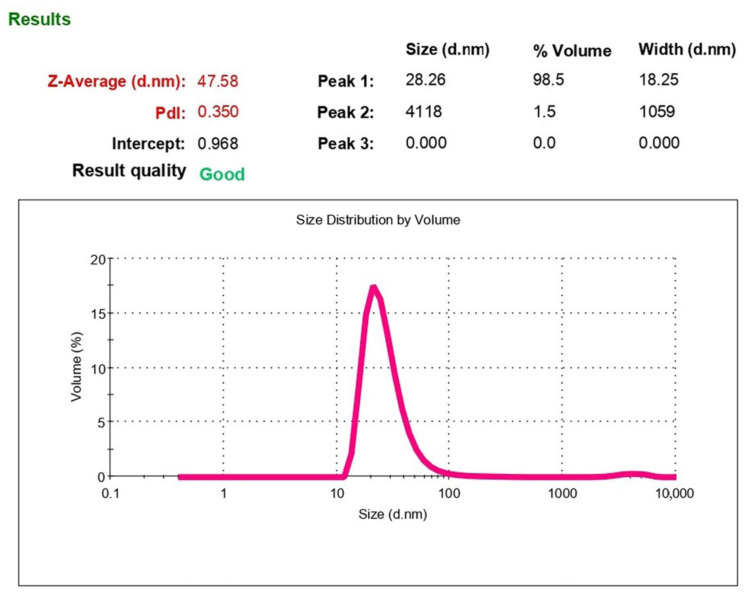
Characteristic particle diameter distribution (polydisperse analysis by volume).

The size of phospholipid nanoparticles was determined using laser correlation spectroscopy. Figure 5 shows the characteristic particle size distribution for a sample of PhTNS.

Figure 5 shows that in monodisperse analysis, an average particle diameter (Z-Average) is 47.58 nm. However, the PI has a rather high value of 0.36, which is due to the presence of several fractions of particles with different sizes. Therefore, it is more correct to estimate the particle size using polydisperse analysis. Figure 5 also presents data on the distribution of particles by volume, indicating the average particle size of this fraction and proportion of volume occupied by particles of this fraction compared to the total volume occupied by all particles. As can be seen, 98.5% of nanoparticles have a diameter of 28.26 nm and only 1.5%—4118 nm. The percentage of large particles shifts the maximum of the monodisperse distribution curve (Z-Average) toward particles with a larger diameter (up to 47.58 nm). 

Thus, the rehydrated emulsion of PhTNS contains more than 98% of particles with a diameter of about 30 nm and about 2% of larger particles.

After rehydration of the freeze-dried samples in the resulting emulsion of PhTNS, the particle diameter corresponded to the data obtained for the parent drugs. 

#### 3.5.2. Transmission Electron Microscopy

Micrographs of aqueous dispersions of nanoparticles were obtained using transmission electron microscopy (TEM) for a more detailed study of the phospholipid nanoparticle size and structure.

**Figure 6:**
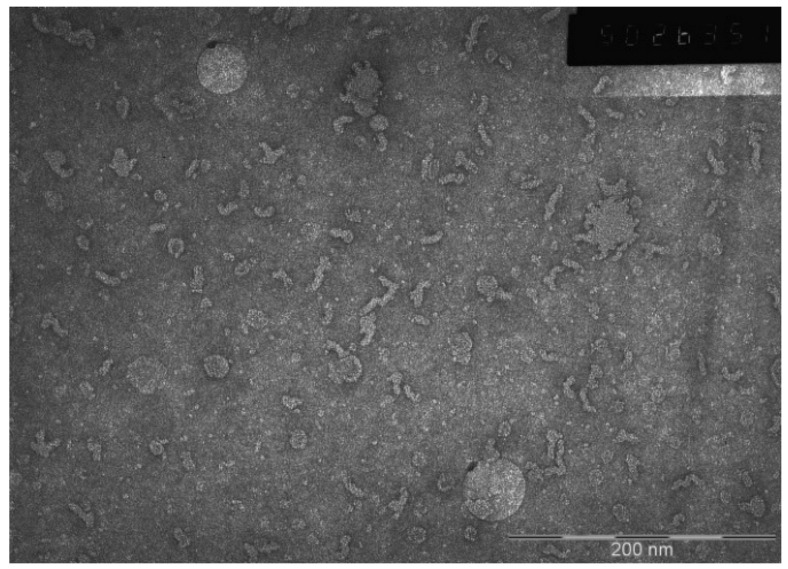
Electron micrograph of a phospholipid nanoparticle sample.

Figure 6 shows micrographs of phospholipid nanoparticles. In the photo, one can see discrete particles that are predominantly spherical and/or rod-shaped. 

Previously, a combination of two methods—small-angle neutron scattering in the range of PhTNS concentration in heavy water of 5–25% by weight and small-angle scattering of X-ray synchrotron radiation in several concentrations in water—was used to determine the single-layer vesicular structure of phospholipid transport nanosystem (PhTNS) [23,24]. It was found that the size of vesicles decreased with an increase in the maltose concentration and was about 27.2 + 0.2 nm at a concentration of 20%.

Thus, the data confirm the results obtained previously using laser correlation spectroscopy.

#### 3.5.3. Electrokinetic Potential (Zeta Potential (ζ))

The stability of particle aggregation in solution is characterized by the zeta potential (ζ), an indicator that evaluates the mutual influence between a dispersed medium and a dispersed particle. The zeta potential is an important indicator of the particle surface charge and can predict and control the stability of colloidal suspensions or emulsions. Also the zeta potential largely determines the pharmacokinetics of the drug embedded in phospholipid nanoparticles and depends on the composition of lipids and their headgroup charges [22]. 

**Figure 7:**
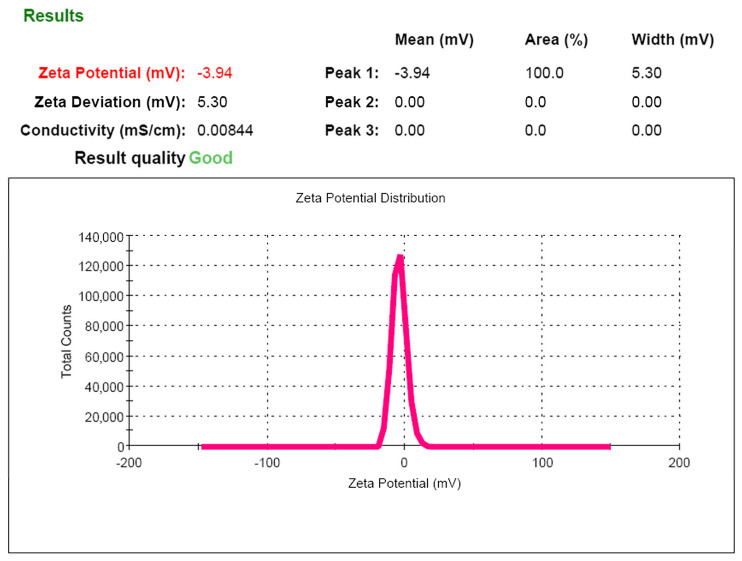
Zeta potential of phospholipid nanoemulsion.

Particles with zeta potentials more positive than +30 mV or more negative than −30 mV are normally considered stable [25]. The ζ-potential value for the system developed was −3.9 ± 1.1 mV. The obtained absolute value of the ζ-potential (Figure 7) indicates the low electrostatic stability of phospholipid nanoparticles in solution and confirms the need to remove the dispersion medium from the solution for long-term storage of the product.

Thus, the Institute of Biomedical Chemistry (IBMC, Moscow, Russia) has developed a laboratory technology for obtaining a phospholipid nanosystem in a freeze-dried form with an extremely small size of particles. The system developed retains its characteristics after rehydration and remains stable during long-term storage (up to 3 years) at a temperature of 25 °C.

### 3.6. Incorporation of Drugs

Incorporation of medicinal compounds in phospholipid nanoparticles is one of the key problems in the development of medicines equipped with transport systems. 

There are techniques for embedding or binding of a water-soluble drug that can be used for both micelles and liposomes. The point is that phosphatidylcholine, the main component of phospholipid nanoparticles, is a zwitter ion by its chemical nature. This means that the polar part of its molecule carries some “excessive” negative charge due to the phosphate group and some “excessive” positive charge due to the nitrogen of choline. These properties can be used to bind a particular drug to the hydrophilic surface of the “phospholipid matrix”, depending on its chemical nature and at appropriate pH values. In the case of liposomes, such interaction occurs both in the internal hydrophilic region of liposomes and in the external one [26]. In the case of micelles, such interaction occurs only on their outer surface [27].

When selecting optimal technological conditions for the maximum incorporation of drugs in phospholipid nanoparticles, the physicochemical properties of both the drug itself and phospholipid particles should be taken into account. The developed technology makes it possible to obtain phospholipid nanoparticles with both hydrophobic and hydrophilic compounds embedded.

A significant number of already existing drugs, as well as those under development, are insoluble in water, i.e., hydrophobic. Because of poor solubility, their bioavailability is usually extremely low. To increase the efficacy of such drugs, it is necessary to overcome the above problem by modifying the molecule or creating new soluble dosage forms. One solution is the supply of hydrophobic medicinal compounds to organs and tissues using transport systems. Liposomes derived from phospholipids were among the first transport systems of biologically active compounds. Several liposomal drugs have already found practical application, in particular in oncology [28].

The PhTNS production technology described above became the basis for the development of the drug incorporation method into phospholipid nanoparticles. Soy phosphatidylcholine (Lipoid S100) was selected as a parent component for the production of PhTNS. It has been shown that a highly dispersed nanosystem obtained on the basis of soy phospholipid is a universal system for transporting a number of biologically active compounds, both fat-soluble (hydrophobic) and water-soluble (hydrophilic). 

**Figure 8:**
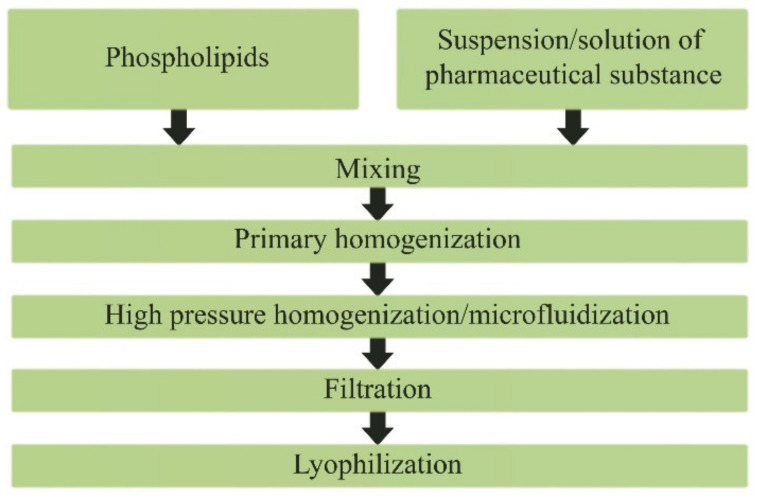
Scheme of obtaining the lyophilized form of phospholipid nanodrugs.

Based on the screening of drugs with different mechanisms of action, we analyzed the possibility of their incorporation into PhTNS, determined the basic principles and developed a technological scheme for obtaining medicinal compositions with a given particle size. We also developed a method to determine the percentage of drug incorporation into phospholipid nanoparticles. The latter parameter is crucial, since the therapeutic efficacy of the resulting dosage form depends significantly on the amount of drug incorporated. The scheme for obtaining the freeze-dried form of nanodrugs is shown in Figure 8.

Table 2 contains physical characteristics: particle size, zeta potential, content of drug and percentage of drug in nanoparticle of phospholipid nanoparticles thus obtained, both free and with drugs embedded (doxorubicin, indomethacin, umifenovir, diclofenac, budesonide, prednisolone, chlorine e6).

As can be seen from Table 2, incorporation of drugs into phospholipid nanoparticles obtained on the basis of soy phosphatidylcholine did not significantly change the final particle size, which was in the range from 8.4 to 28.9 nm. At the same time, when a drug was embedded in the PhTNS, the aggregation stability of the nanosystem tended to increase.

**Figure 9:**
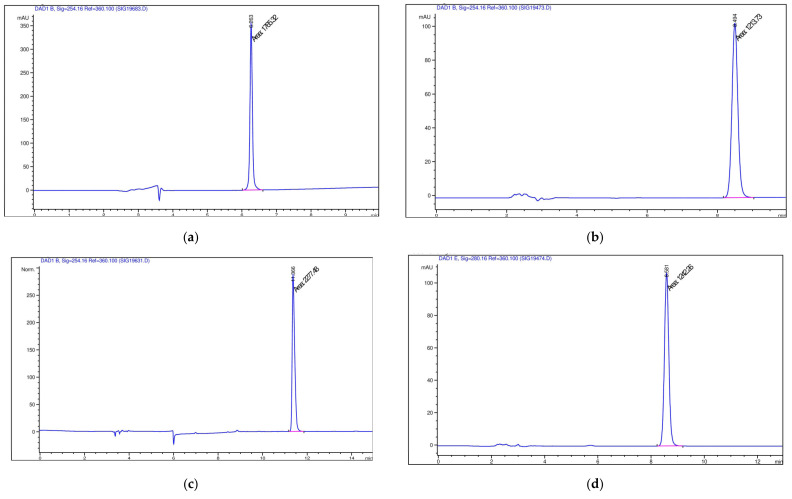

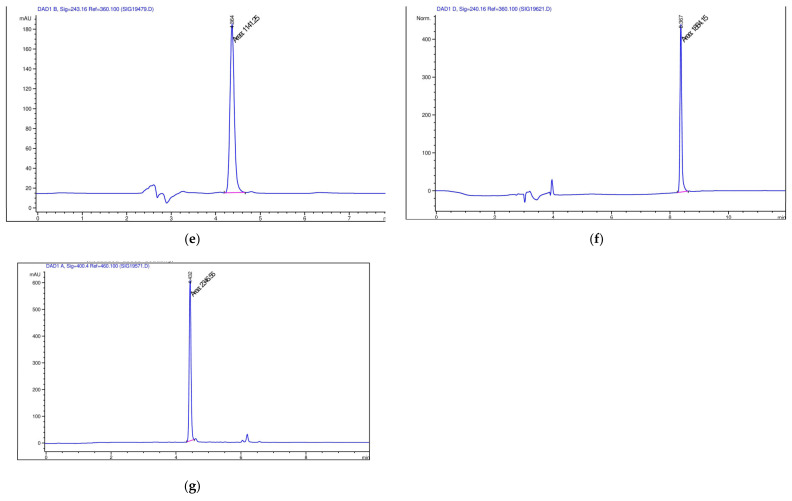
Chromatograms obtained by HPLC of (**a**) doxorubicin; (**b**) indomethacin; (**c**) umifenovir; (**d**) diclofenac; (**e**) budesonid; (**f**) prednisolon; (**g**) chlorine e6 (blue line- the chromatographic profile, pink line – the width of the main chromatographic peak at the baseline).

Chromatograms obtained by HPLC for drugs are shown in Figure 9.

**Figure 10:**
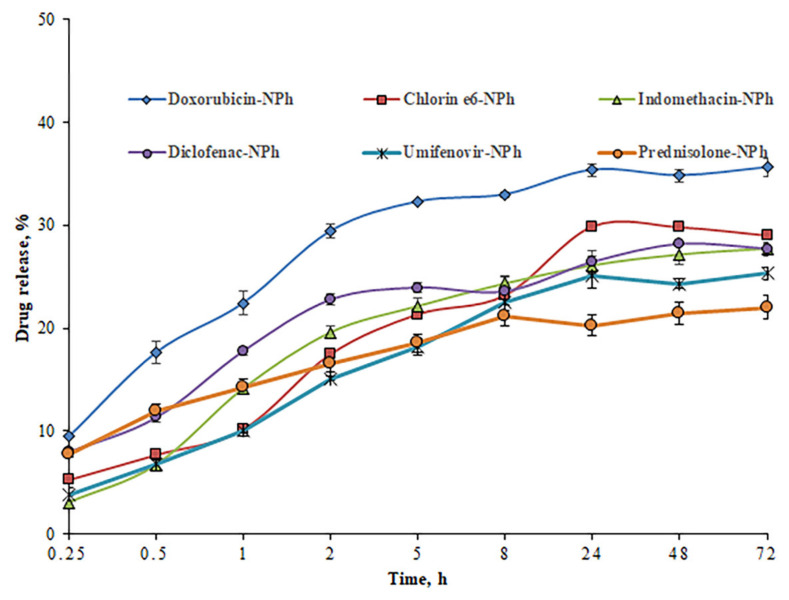
The degree of drug release (concentration of 1 mg/mL) from nanoparticles, depending on the incubation time in PBS (pH 7.4) (drugs embedded in PhTNS are denoted as «Drug-NPh»).

Of great interest is a sudden release or drug leakage into the bloodstream due to the instability of the nanocarrier, which may reduce the therapeutic effect. This poses a serious problem for the drug delivery system in vivo. The stability of drug compositions was assessed by drug release using the method of dialysis at pH 7.4 (Figure 10).

The study has shown a gradual release of drugs from the nanocarriers. However, after 72 h, the percentage of drug release for most samples were 20–29%. Only for the sample with doxorubicin, this parameter was slightly higher compared to other samples, but did not exceed 36%. The maximum drug release was observed after 24 h. During the next two days, the percentage of drug release practically did not change. Thus, the compositions obtained are stable under normal physiological conditions (pH 7.4). Because of a stronger interaction between the drug and the nanocarrier, most of the drugs remained in the phospholipid nanoparticles for three days, which proves the possibility of a prolonged retention time of drugs in the body.

## 4. Conclusions

We have developed a technology for obtaining a stable phospholipid transport nanosystem (PhTNS) with an extremely small particle size in a freeze-dried form with reproducible characteristics. Using laser correlation spectroscopy methods, the particle size was determined to be near 30 nm, which was also confirmed by the methods of small-angle scattering of neutrons (SANS) and X-ray (SAXS). Nanoparticles are single-layer vesicles, the size of which is inversely related to the concentration of maltose in an aqueous solution. Application of transmission electron spectroscopy has shown that PhTNS consists of discrete particles that are predominantly spherical and/or rod-shaped.

We have obtained and characterized the phospholipid nanoparticles loaded with drugs of various chemical structures and pharmacotherapeutic groups, the inclusion rate of which was at least 92%.

The in vitro release of drugs embedded in PhTNS has been also studied at a physiological pH value. Thus, the present study showed the stability of drug-loaded formulations in PhTNS for 72 h, and a more sustained release profile under normal physiological conditions (pH 7.4).

## Figures and Tables

**Table 1:** Chromatography conditions for particular drugs.

Drug	Elution	Detection (Signal/Reference, nm)	Retention Time, min
Doxorubicin	30–90% B—0–10 min	254/360	6.3
Indomethacin	60% B—10 min	254/360	8.5
Umifenovir	0–60% B—0–5 min 60% B—5–15 min	254/360	11.4
Diclofenac	60% B—13 min	280/360	8.6
Budesonid	75% B—8 min	243/360	4.4
Prednisolon	10–60% B—0–5 min 60% B—5–12 min	240/360	8.4
Chlorine e6	60–99% B—0–1 min 99% B—1–10 min	400/460	4.4

**Table 2:** Characteristics of PhTNS-based drugs.

Drug	Particle Size, nm	Zeta Potential, mV	Content of Drug, mg	Percentage of Drug in Nanoparticles, %
Phospholipid nanoparticles	28.9 ± 1.2	−3.9 ± 1.1	-	-
Doxorubicin	19.2 ± 3.1	6.5 ± 0.8	13.0	96
Indomethacin	21.9 ± 1.9	−12.9 ± 0.6	23.8	95
Umifenovir	8.4 ± 2.6	43.8 ± 1.8	24.6	98.3
Diclofenac	12.9 ± 3.0	−46.6 ± 1.5	23.0	92
Budesonid	22.4 ± 4.6	−1.6 ± 0.2	5.0	100
Prednisolon	18.3 ± 4.1	−4.6 ± 1.3	14.7	98
Chlorin e6	21.2 ± 3.3	−25.5 ± 4.0	12.3	98.4

## Data Availability

Not applicable.
